# How does a targeted active labour market program impact on the well-being of the unemployed? A concept mapping study on Barcelona “Employment in the Neighbourhoods”

**DOI:** 10.1186/s12889-020-8441-2

**Published:** 2020-03-17

**Authors:** Vanessa Puig-Barrachina, Davide Malmusi, Lucía Artazcoz, Xavier Bartoll, Eva Clotet, Imma Cortès-Franch, Lorena Ventura, Ferran Daban, Èlia Díez, Carme Borrell

**Affiliations:** 1 Agència de Salut Pública de Barcelona, Pl. Lesseps, 1, 08023 Barcelona, Spain; 2 CIBER de Epidemiología y Salud pública (CIBERESP), Madrid, Spain; 3 Ajuntament de Barcelona, Barcelona, Spain; 4 Institut d’Investigació Biomèdica (IIB-Sant Pau), Barcelona, Spain; 5 Universitat Pompeu Fabra, Barcelona, Spain; 6 Barcelona Activa, Barcelona, Spain; 7 Program in Methodology of Biomedical Research and Public Health, Universitat Autònoma de Barcelona, Barcelona, Spain; 8 Program in Biomedicine, Universitat Pompeu Fabra, Barcelona, Spain

**Keywords:** Active labour market programs, Unemployment, Barcelona City (Spain), Deprived neighbourhoods, Gender, Quality of life, Concept mapping

## Abstract

**Background:**

The “Employment in the neighbourhoods” program is an innovative, tailor-made Active Labour Market Program that has been implemented in 12 neighbourhoods in Barcelona (Spain). Its goal is to get people from deprived, high-unemployment neighbourhoods back to work. The aim of this study was to describe the effects of the program on participants’ quality of life, and identify the mechanisms underlying these effects, according to their own perception and the perception of technical staff who assisted them.

**Methods:**

We used Concept Mapping, a mixed methods approach combining qualitative and quantitative analysis, to develop a conceptual map of the participants’ and technical staffs’ perceptions about changes in the participants’ quality of life. Data collection occurred within the generation and structuring steps where participants brainstormed answers to a focus question, and then rated and sorted the responses. To create maps, we used Concept Systems Incorporated software, which conducted two main forms of analysis, a multidimensional scaling analysis, and a hierarchical cluster analysis.

**Results:**

Study participants reported several positive effects on mental health and emotional wellbeing, including self-esteem and empowerment, and considered that this was achieved through strengthened social networks, skills acquisition, emotional coaching, and personalized technical assistance. They also described some negative impacts, mainly related to the labour market situation. We observed marked gender differences in the discourses of program participants.

**Conclusions:**

The results obtained have allowed us to identify different perceived effects and mechanisms by which the “Employment in the Neighbourhoods” Active Labour Market Programme can influence quality of life of participants from the most deprived areas of Barcelona.

## Background

### Introduction

Since 2008, unemployment has risen sharply in many European countries due to the global economic crisis and the prolonged recession. In addition, the European Union imposed a political agenda of austerity on Southern European countries in exchange for financial assistance, and this contributed to exceptionally high unemployment rates in Spain, Italy, Greece, and Portugal [1]. Unemployment has multiple striking social effects, including poor physical and mental health, and low subjective well-being and life satisfaction [2], and is one of the most important social determinants of health inequalities [3].

During the 1990s, and for economic reasons, many countries introduced Active Labour Market Programmes (ALMPs) in an effort to reduce unemployment [4]. ALMPs aim to reintroduce participants into the labour market through vocational training courses, improved job search skills, work experience (internships and subsidized jobs), and behavioural programmes, among others [4, 5]. In terms of health and wellbeing, ALMPs are thought to ease the negative effects of unemployment through different psychosocial mechanisms [6].

A recent review showed that most studies that assessed the health effects of ALMPs focused on ALMPs implemented in Anglo-Saxon and Nordic countries [6]. However, the effects of the recent economic crisis were most strongly felt in Southern European countries, but to date little is known about the health impact of ALMPs in these countries. To our knowledge, only one study has evaluated the health effects of an ALMP implemented in Spain [7], and no similar study has been conducted in other Southern European countries [6]. In addition, few reports on this topic discuss the mechanisms involved in this improvement.

“Employment in the Neighbourhoods” (EiN) is an ALMP implemented in Barcelona, Spain, that aims to reduce social inequalities through labour market insertion of people living in neighbourhoods with the highest unemployment rates in the city, and who have the greatest material needs. A pre-post intervention study using quantitative research methods showed improved mental health among participants, while self-perceived health status remained stable or worsened [8].

The general objective of this paper is to describe the effects of EiN on quality of life and the mechanisms involved, according to the perception of the program’s participants and the technical staff who assisted them. Technical staff members are those professionals who accompany participants through the entire process as personal guidance counsellor.

### Unemployment and health: pathways and mechanisms

Unemployment has been shown to cause poor health through material and psychosocial pathways. Material effects are primarily due to poverty resulting from lost income and financial strain. Psychosocial factors include the loss of non-economic resources provided by employment. According to Jahoda’s “latent functions” theory [9], while people primarily engage in paid work to earn their living (the manifest function of employment), they also profit from five latent functions that are unintended by-products of employment, namely time structure, social contact, collective purpose, social identity/status, and activity. Moreover, uncertainty about one’s future work situation is also a stressor that leads to physiological changes, risky health behaviours, and consequently, poorer health [10, 11].

### ALMPs and health: effects, pathways and mechanisms

ALMPs are thought to ease the negative effects of unemployment on health and wellbeing by promoting participation in work-related activities [12]. ALMPs alter unemployed people’s environment by providing an opportunity to meet, socialize, and strengthen social networks, and offer daily activities, routine and structure [12]. In terms of status, vocational training courses may provide the perception of involvement in a legitimate alternative status to employment, namely education status [12]. Improved job search and related skills, such as writing a *CV* and learning to respond in a job interview, could increase one’s feeling of control over one’s life course, and workplace participation through work experience and training in a regular market could reduce the psychosocial need for employment by providing a more employment-like life situation [12].

Based on the above theoretical knowledge, we can generally expect involvement in an ALMP to have a positive impact on health and wellbeing compared to open unemployment situations (i.e. being unemployed not being enrolled in any ALMP). A recent scoping review including 36 quantitative and qualitative studies conducted from the 1990s to the present day, showed mainly positive health impact of ALMPs in high-income countries. 80.6% of the studies included in the review found a positive impact on health or quality of life, and the rest showed no effects [6]. Of all types of the ALMPs implemented, the only type that always showed a positive effect on mental health was the one that offered job search assistance accompanied by a psychological component, such as improving self-confidence, self-image, or self-efficacy [6]. This result was the same for the different countries where it was implemented (Australia, EUA, Finland). The rest of types showed controversial results. The heterogeneity of contexts where these programs were implemented and the heterogeneity of programs’ content could partly justify these controversial results. For example, the diverse effects of such programs on health in the same context are evidenced by Strandh et al. [12], who showed that in Sweden at the end of the 90’s, unemployed individuals involved in workplace participation reported better mental health than those in open unemployment; however, those involved in vocational training did not experience improved mental health.

Few of the studies mentioned above explored the mechanisms underlying the health changes caused by participation in the ALMP. Quantitative evaluation of programs offering job search assistance and psychological component (e.g. inoculation against setbacks or increasing self-efficacy) reported positive results in terms of Fryer’s theory of gaining mastery and control over one’s life course [13]. Sense of mastery and a general increase in labour market engagement could be related to improved mental health [14]. This type of program has only been implemented in the USA [15], Finland [14] and Australia [16]. Similarly, qualitative evaluation of the programs offering job search activities to young people in Finland [17] reported positive results in terms of Jahoda’s model of latent function, by providing a structure of life [9]. The study showed how this type of programs help transform unemployment into a meaningful pattern of time use, especially through work-like activities or education. Moreover, in line with the idea of sense of mastery and economic control, participants reported seeing new possibilities in the labour market, and having economic support from the program.

### Contextual factors matter

The impact of ALMPs may depend on various social and political factors, such as the “model of activation” [18], gender roles, family as a social institution, and the culture of work. “Model of activation” refers to the policy strategy aimed at reducing unemployment, as well as the corresponding social protection system for the unemployed. There are two major activation models, “work-first” or “liberal type” and the “universalistic type”. In “work-first” or liberal type, the role of ALMPs and social policies is limited to inciting individuals to seek work, providing quick information and simple matching services, investing in short-term vocational training, inciting people to be as active as possible throughout life, and accepting any job on offer [18]. On the other hand, the “universalistic type” of activation guarantees relatively high standards of living for unemployed people and does not push them to accept any job in the market [18]. The liberal model is typical of Anglo-Saxon countries, and the universalistic of Scandinavia, while most other countries, such as continental and Southern European countries, oscillate between these two.

For example, in Germany “One-Euro-Jobs” is an ALMP based on subsidized employment that includes coercive elements in line with those of the liberal model. Individuals who participate in this program did not perceive improved integration in comparison to those in open unemployment [19]. The authors argue that paternalistic programmes that work through directive methods and impose unilateral obligations can affect individual’s interpretations of their role and status in society.

Gender and family also have a fundamental influence on the unemployment experience, with some authors suggesting that non-work is less damaging in societies where there is a stronger emphasis on family life [20, 21], such as for example the Southern European countries. In these countries, where the Welfare State is weaker in terms of universal social policies, additional material and non-material support is provided by the family. In that case, we could hypothesise that ALMPs are not considered to be as important for social support as in other countries, if the traditional family plays its role. On the other hand, in these countries, where the Welfare State is less developed, women are still most responsible for informal care, while men still play the breadwinner role, at least in archetypal terms. It has been shown that in countries where gender roles are still traditional and differentiated, unemployment has a greater effect on males, because it impairs their individual/social identity [3]. In that sense if ALMPs contribute to supporting this identity, they could play a very important role among unemployed men.

In terms of culture, it has been proposed that the moral virtues of work characteristic of the Protestant ethic are central to the Nordic work ethic. In this culture, work provides good role models and stimulates good behaviour [20]. Work ethics has an important influence on the significance of employment, probably on the unemployment experience, and thus, in the meaning and effects of ALMPs.

In this sense, it is particularly relevant that most evaluations of ALMPs were conducted in Anglo-Saxon and Scandinavian countries, and only one study has been carried out in Eastern Europe countries [22] and one in Southern [7]. Thus, it is at least reasonable to question whether the health outcomes and the underlying mechanisms of ALMPs are transferable to different contexts. There is a lack of empirical and theoretical studies addressing these issues.

### ALMPs in Spain

Spain is one of the countries that spends the smallest fraction of its GDP on ALMPs, which is thought to be an important determinant of health and wellbeing among unemployed people [23]. Spain’s unemployment benefits are less generous than Nordic ones, and unlike the liberal model of activation, unemployment benefits are not compulsorily linked to ALMPs. In terms of gender and family, Spain is characterized by a strong “familialism”, with a family solidarity model based on an asymmetric gender division of work and low female participation in the labour market. Spain has residual family policies with lack of support for families, making them rely on unpaid work [24]. To our knowledge, ALMPs in Spain have not been evaluated in detail, with the exception of one study [7] that evaluated the effects of the Madrid Government’s minimum income program (IMI) on health and wellbeing.

### “Employment in the Neighbourhoods”: an ALMP to reduce social inequalities in Barcelona, Spain

In 12 neighbourhoods of Barcelona (Catalonia, Spain), an innovative tailor-made ALMP called “Employment in the Neighbourhoods” (EiN) (“Treball als Barris” in Catalan) was implemented to get people from deprived, high-unemployment neighbourhoods back to work, and to promote socioeconomic revitalization of these neighbourhoods. This program is part of a broader initiative, “The Neighbourhoods Law”, launched in 2004, to reduce social inequality in Catalonia by improving the quality of life in specific neighbourhoods primarily through urban renewal projects (DPTOP, 2009).

EiN is run by Barcelona City Council through its employment promotion agency, “Barcelona Activa”, and is partly financed by the Catalan Employment Service (Autonomous Government of Catalonia). Enrolment in the program is voluntary, and participants are required to be registered as a job seeker and to reside in one of the neighbourhoods where the program is implemented. The program prioritises people at risk of exclusion, i.e. those aged > 50 years who are long-term unemployed, those under 35 years who left school early, those with low professional qualifications, women who have suffered gender violence, women who want to re-join labour market, and people with physical disabilities.

The program enrols 1500–2000 people per year, most of whom have a basic or no formal education. A high percentage of participants are foreigners, mostly non-EU nationals, and most are long-term unemployed who are not eligible for contributory unemployment benefits. Unemployed persons who wish to join the program first attend a personal interview with a trained professional to evaluate their occupational status and develop a tailored job-search plan. The duration of the program depends on each specific job-search, and can range from a few weeks to a few months, with a maximum of one year.

The program’s methodology is based on customized integration pathways that combine several insertion measures. It mostly provides job search guidance and professional skills training to increase employability, and promotes social and professional integration support for people with special difficulties, considering the specific needs and potential of each neighbourhood and working in close coordination with local associations and the main social agents (See Table S1 for specific actions).

Thus, we expect EiN to ease the negative effects of unemployment on participants’ health and wellbeing by providing an opportunity to socialize and strengthen social networks, and, for those who participate for at least several weeks, a routine and structure through daily activities. More specifically, we expect them to have greater control over their life course through job search activities, the perception of involvement in a legitimate alternative to employment through professional skills training, and a reduction of the psychosocial need for employment through social integration support.

In order to describe the effects of EiN on quality of life and the mechanisms involved, we developed two conceptual maps with a cluster approach based on the perceptions of the participants and technical staff using the Concept Mapping technique.

## Methods

The study uses a phenomenological design to explain how people describe social phenomena through their actual experiences [25]. We used Concept Mapping (CM), a mixed methods approach that combines qualitative and quantitative analysis [21]. Through CM we developed a conceptual map based on the perceptions of the participants and technical staff of EiN; EiN managers were excluded. CM has proven to be a useful method for evaluation, which can be used directly with participants to map how programs have affected them [26]. The study was conducted between March and July 2016 on Barcelona Activa premises.

The sample design followed a theoretical plan that determined the typologies of profiles of the sample units to obtain as much discursive variability as possible, thereby guaranteeing that different conceptual meanings of the study phenomenon are collected [25]. We constructed typologies according to the characteristics we considered would influence the meaning of the phenomenon under study. In the case of participants, we also introduced two segmentation criteria, their employment situation after the program (unemployed vs working or having worked for at least three months), and their gender. We decided to preserve the gender stratification during brainstorming sessions due to possible differences in communication patterns between genders. In mixed-gender public gatherings, men are known to spend more time talking than women and are more likely to interrupt, such that women generally have less confidence in their ability to make arguments. Moreover, due to the gender division of labour, which is still present to a lesser or greater extent in most societies, the mechanisms that link the intervention to health and well-being may differ between men and women. Thus, we created four groups of EiN participants: unemployed females, employed females, unemployed males, and employed males. Within each of the above-mentioned groups of participants, we aimed to achieve heterogeneous groups in terms of country of birth and neighbourhood of residence. Professionals and program users came from the nine decentralised job-search information points located in different neighbourhoods of Barcelona.

In order to recruit the participants, two members of the research team went to the premises of “Barcelona Activa” to ensure confidentiality of personal data, where they obtained the complete list of participants in the EiN during 2015. Participants who were still enrolled in the program in 2016 were discarded. The lists were obtained according to sex, neighbourhood of residence and country of birth. We randomly called person number 1, then 11, then 21 and so on for each stratum, e.g. females born in Spain from neighbourhood number 1. During the telephone call, in addition to explaining the study and inviting them to participate, they were asked whether, after carrying out the programme, they had worked for at least three months. Recruitment stopped when 20 participants were obtained for each group. The ideal size for the first phase of the CM, brainstorming, is about 10 participants for each group, and we expected a 50% loss.

Regarding the group of technical staff, we invited to participate, all who were currently working at the time of the research study or had recently been working for a long period in EiN.

### Concept mapping methodology

Concept mapping comprises three data collection phases: brainstorming, idea structuring, and participatory interpretation [26]. Data analysis is conducted between idea structuring and participatory interpretation. Qualitative data are collected during the brainstorming and interpretation steps, and quantitative analysis is then used to construct the maps in order to understand these ideas.

### Brainstorming

At the outset of the project, researchers agreed on the following focus statement for the brainstorming activity: “One way the Employment in the Neighbourhoods program has influenced my quality of life is …” ; for ALMP technical staff, the focus statement was slightly modified as follows: “One way the ALMP has influenced the quality of life of “Employment in the Neighbourhoods” participants is …” . We held a brainstorming session of approximately 1 h for each of the five groups. Key terms were further clarified; the term “quality of life” was defined as a multidimensional construct that includes emotional wellbeing, interpersonal relations, material wellbeing, personal development, physical wellbeing, self-determination, social inclusion, and rights [27]; influence was defined as either positive or negative. The research team encouraged discussions on negative experiences, and highlighted that staff’s work would not be evaluated according to the effects on participants’ health, in order to avoid reporting mostly positive effects.

Quality of life dimensions were considered as a starting point for the discussion to describe effects and also mechanisms. Quality of life contains different dimensions that can be considered mechanisms within a chain of causality when considering unemployment situation and the effects of ALMP. The research team did not introduce technical aspects, such as which items could be considered effects and which considered other mechanisms. However, as moderators of the brainstorming session, when two items were linked as a causal chain, we asked participants to verify this relationship.

A minimum of two people from the research team conducted the sessions. One moderated the session by trying to allow everyone to participate and express their opinions, covering most of the dimensions of the concept of quality of life, and facilitating both positive and negative effects. The other took notes on the board and commented on the ideas. All sessions were audio recorded and photographs were taken.

We collected participants’ demographic characteristics, including age, gender, educational level, employment status, both currently and after having participated in EiN, and the number of months of employment in case they were working. For technical staff the questionnaire collected data on age, gender, educational level, the number of months/years employed as EiN professionals, and the number of years of experience as occupational staff. Participants received a 10-journey-card for public transport as compensation and to overcome the participation barrier.

Once all brainstorming sessions were completed, the research team reviewed the final statements to avoid repetition. Two lists of statements were created, the first from ALMP participants and the second from technical staff. We systematically condensed all brainstorm responses from all participants, i.e. the lists from unemployed men, unemployed women, employed men and employed women, into a unique list of 53 statements; we condensed the responses from technical staff into 43 unique items.

### Idea structuring (sorting and rating)

Approximately 2–3 weeks after the initial session, a second session of approximately 2 h was held for each group to sort and rate responses. Separate sessions were organized for EiN participants and technical staff. During this session, participants worked individually, and 2 to 4 researchers were present to assist with literacy issues or other questions. First, participants were asked to sort the statements presented on cards into groups or themes that made sense to them, and to name each pile created. ALMP participants sorted their own list of 53 statements, and technical staff sorted their list of 43. Then, ALMP participants were given a rating sheet with the same corresponding statements and asked to rate the importance in relation to quality of life of each corresponding statement on a 5-point Likert-type response scale: from [1] little importance to [5] extremely important. In addition, participants were asked to rate the frequency with which they have experienced the statement from [1] never occurred to [4] occurred frequently. Technical staff were also given a rating sheet to rate importance of their statements on the same Likert-type response scale; and the frequency of occurrence according to their professional knowledge, from [1] infrequent to [5] very frequent.

### Data analysis

Using data from the second session, we generated maps and rankings using software from Concept Systems Incorporated [28]. To create the maps, the software conducts two main forms of analysis, first a multidimensional scaling (MDS) analysis followed by hierarchical cluster analysis [26]. The analysis begins by transforming the sorted piles of statements into a symmetric binary matrix of similarities for each participant. A total similarity matrix is obtained by summing across the individual matrices. This similarity matrix shows the number of participants who sorted each pair of statements together. As it is impossible to visualize k-dimensional space, a multidimensional scaling (MDS) algorithm is used to transform the high-dimensional data into two-dimensional space, while trying to preserve the pairwise distances as much as possible [29]. The extent to which the original relative distances are preserved in the two-dimensional space is measured by the “stress” index. The more the MDS algorithm successfully preserved pairwise distances, the lower the stress index. For concept mapping projects, stress values usually range from 0.205 and 0.365 [26]. High stress values imply greater complexity in the similarity matrix than can be represented well in two dimensions, and that there was considerable variability in the way participants grouped the statements, or both. Stress index values were calculated for each of the point maps. Another issue considered when constructing the map was the choice of cut-off level. The cut-off level is used to refine the analysis results by identifying instances of “outlying” sorts, on the item level. It is of great value when it is difficult to interpret results with all data included. Thus, cut-off level filters out spurious relationships between statements. Only associations above the cut-off level are mapped. For example, if a value of 2 is specified, this means that if only two or fewer users placed two statements together in a pile, the analysis will treat it as though no users placed the statements together. The choice of a high cut-off level simplifies the map by reducing the number of associations found; a low cut-off level may result in a complex map that is difficult to interpret.

Thus, the result of this step is a two-dimensional map of points, where each statement generated during the brainstorming is represented by a dot and identified by its corresponding number. The position of all statements tends to reflect the computed distances between them. From here, the next step is to form clusters of statements that capture more general themes or concepts. The Euclidian distances between points on the map are then grouped using Hierarchical Cluster Analysis (HCA) to form clusters of statements using Ward’s algorithm [30]. Agglomerative HCA gives as many possible cluster solutions as there are statements. The task for the analyst is to decide the number of clusters that the statements should be grouped into for the final cluster solution. There is no mathematical way to select the final cluster solution. Researchers choose the final cluster numbers solution based on the consistency between the clusters’ contents, their knowledge on the issue, and a group discussion with some participants on the perceived changes. In our case, the final group discussion, also known as participatory interpretation, was carried out with the group of technical staff only. Cluster names were derived from the descriptive labels participants were asked to provide and the research team’s knowledge on the subject [26]. Finally, the average ratings regarding importance and frequency of each statement, and the average ratings for all statements in a cluster were calculated for EiN participants (stratified by sex) and for EiN professionals.

### Participant characteristics

Four groups of ALMP participants were formed from a total of 41 participants (Table 1). These 41 individuals attended the brainstorming session, while only 33 of them attended the sorting and rating session. Most foreign-born participants came from South and Central America, followed by those born in Morocco, and a small representation from Asia and Sub-Saharan Africa. Most participants had elementary or post-elementary education, a few had university-level education, and some were illiterate.

**Table 1:** Demographic and socioeconomic characteristics of participants in all groups

	Women		Men	
	Unemployed (*N* = 7)	Working (*N* = 11)	Unemployed (*N* = 11)	Working (*N* = 12)
**ALMP Participants (*N*** **= 41)**				
**Age**				
Minimum	34	32	32	32
Maximum	57	55	56	62
Median	45	43	39	50
**Country of birth**				
Spain	2	6	3	7
Others	5	5	8	5
**Number of hours in the program**				
Minimum	7	9	3	14
Maximum	86	86	104	116
Median	11	27	23	55
**Technical Staff (*N*** **= 9)**				
**Sex**				
Female	*N* = 8			
Male	*N* = 1			
**Age**				
Minimum	31			
Maximum	45			
Median	43			
**Years of experience as a coach in insertional programs**				
Minimum	5			
Maximum	25			
Median	10			
**Months of experience in “Employment in the Neighbourhoods”**				
Minimum	7			
Maximum	97			
Median	36			

The group of professionals included 9 members of technical staff of EiN. 8 women and 1 man attended the first brainstorming session, and 7 people attended the second session (sorting and rating). Most of the technical staff was trained in psychology, pedagogy, and social work. There was high variability in their time and experience in EiN, from 8 months to more than 8 years, as well as variability in time-experience as labour market coaches.

The numbers and characteristics of participants are shown in Table 1.

## Results

### Influence of the “Employment in the Neighbourhoods” program on quality of life: participants’ perception

**Fig. 1:**
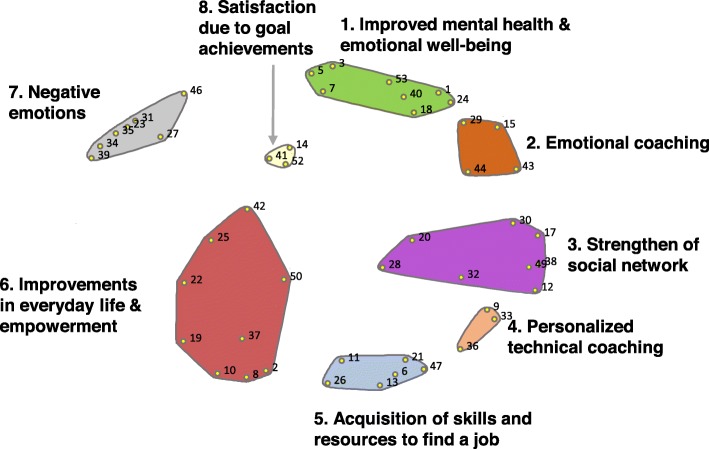
Cluster map derived from participants perceptions of the impact of “Employment in the Neighbourhoods” on their quality of life

A set of statements were generated by each group of ALMP participants representing changes in their quality of life as a result of the EiN program. The four groups of ALMP participants brainstormed a total of 53 statements, which are organized in clusters in Table 2, ordered by frequency and importance rating, and stratified by sex. Figure 1 illustrates the resulting cluster map. Since the similarity between participants’ perceptions resulting in similar items, we constructed a single map for all participants. The final map showed 8 clusters: 1) “Improvement in mental health and well-being”, 2) “Emotional coaching”, 3) “Strengthening of social network”, 4) “Personalized technical assistance”, 5) “Acquisition of skills and resources to find a job”, 6) “Improvement in everyday life”, 7) “Negative emotions”, and 8) “Satisfaction in achieving goals”. The final point plot had a stress index of 0.34, with a similarity cut-off filter of 3.

**Table 2:**
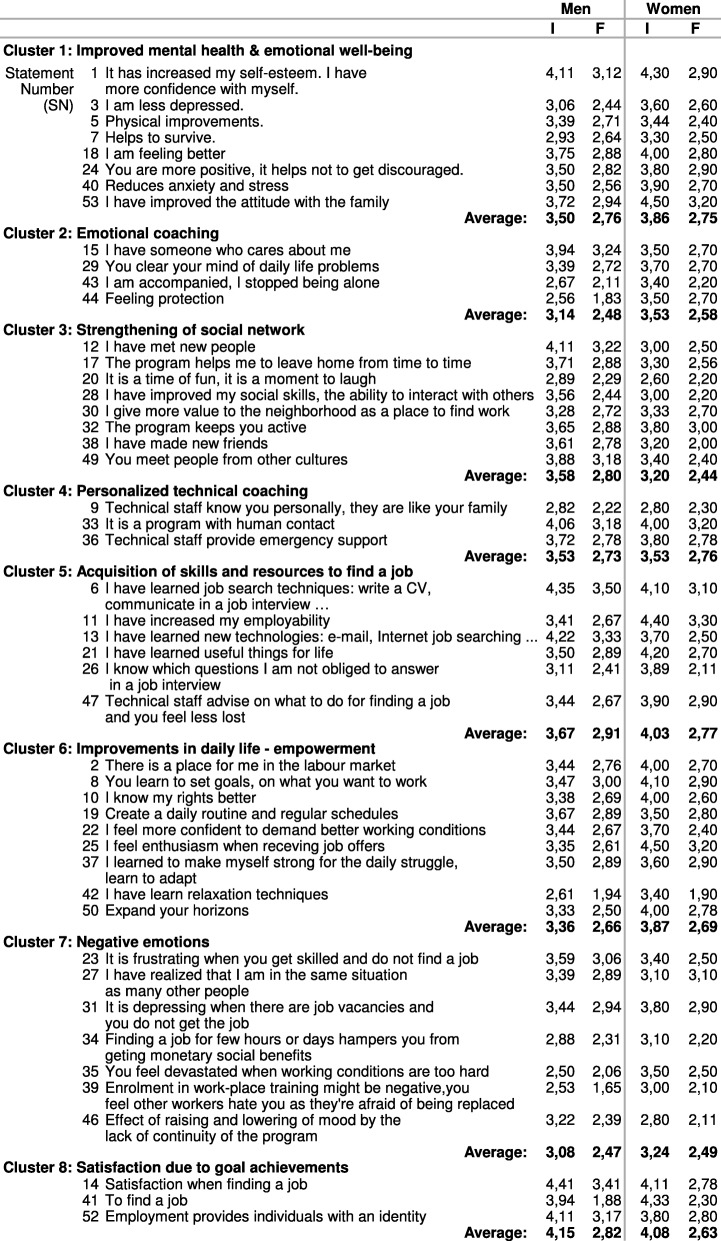
Statements and clusters resulting from group sessions held with EiN participants and their scores based on criteria of importance (I), from [1] little importance to [5] extremely important and frequency (F) according to their own experience, from [1] never occurred to [4] occurred frequently

We observed marked differences between the discourses of males and females during the brainstorming sessions. The female discourse emphasized psychological and emotional improvement (Cluster 1), in both the employed and unemployed groups. Women highlighted the work of the professionals as one of the key factors that influenced their quality of life, with statements such as, “having someone who cares about them” (SN 15), “feeling accompanied” (SN 43), “feeling protection” (SN 44), “technical staff knows you personally” (SN 9), and “technical staff provides emergency support” (SN 36). They related these items with “increased self-esteem and confidence” (SN 1), “decreased depression” (SN 3), and “decreased anxiety and stress” (SN 40). They also reported increased self-esteem and confidence (SN 1, Cluster 1) as a result of the learning process, through courses in new technologies (SN 13, Cluster 5), and interviewing and professional skills, among others (SN 6, Cluster 5). Women highlighted the programs’ ability to reactivate their social networks (Cluster 3). Being enrolled in the program and having someone waiting for them acted as “an obligation to leave home” (SN 17). Increasing their social network had a positive effect on mental well-being, decreasing levels of anxiety and stress (SN 40, Cluster 1). Both groups of women also highlighted increased employability after the program (SN 11, Cluster 5), and having a job (SN 41, Cluster 8) as a key factor for quality of life. However, the women who had found a job emphasised the precarious employment conditions (SN 35, Cluster 7) and the consequent demoralization and loss of mental well-being. During the rating session, women again highlighted, as the most important and commonly experienced items, increased employability (SN 11), an improved attitude with their family (SN 53), and a feeling of enthusiasm when receiving job offers (SN 25).

The **male discourse** during the brainstorming sessions focused on the importance of finding a job (SN 41) through the program as the main source of well-being. Pathways to improved quality of life were derived from being employed. Economic stability was recognized as a source of material well-being, i.e. meeting living standards such as housing. Men highlighted having a job as a source of identity (SN 52, Cluster 8), and emphasized that they had a better relationship with their families when they had a job (SN 53, Cluster 1), due to their role of breadwinners. According to them, their value as a family member increased when they could provide economic resources to their families. Moreover, going to work helped improve their psychological well-being, as they felt calmer and were able to forget about other problems while working. Consistent with this, one of the most important ideas among males was the feeling of frustration when they did not achieve their objective (SN 23, Cluster 7).

Notwithstanding their main objective, they appreciated other benefits derived from being enrolled in the program. They highlighted the need to be active, to occupy their time and have a structured day (SN 32, Cluster 3), which was met by the program. Another main idea highlighted by men was the program’s lack of continuity (SN 46, Cluster 7). They appreciated the program’s positive effect on their emotional well-being but noted that this was not stable over time. Some of them referred to the 6 months duration of subsidized jobs, and highlighted the need for longer occupation plans and continued technical support and emotional counselling. During the men’s rating session, the most important and commonly experienced items were learning job-seeking techniques (SN 6), satisfaction when finding a job (SN 14), and learning new technologies (SN 13).

All participants (men and women) emphasized emotional proximity as a specific and important aspect of the EiN program, compared to other employment integration programs they had previously attended. According to all groups, this was the program’s most important added value: having a person of reference who knows them personally and could help them when needed.

### Influence of the “Employment in the Neighbourhoods” program on quality of life: perception of the technical staff

**Fig. 2:**
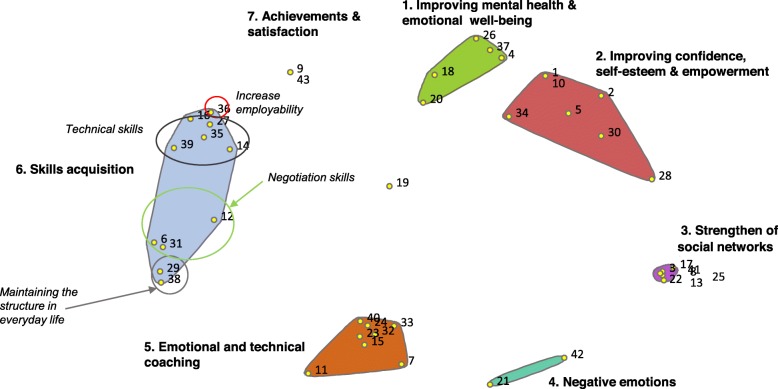
Cluster map derived from technical staff perceptions of the impact of “Employment in the Neighbourhoods” on their quality of life

The technical staff group generated a total of 43 statements on their perception of how participants’ quality of life is influenced by the program. The Point Plot achieved an optimal stress index of 0.293, with a similarity cut-off filter of 1. The concept mapping analysis mapped the 43 statements which are organized in clusters in Table 3, ordered by frequency and importance rating. The resulting cluster map is illustrated in Fig. 2.

**Table 3:**
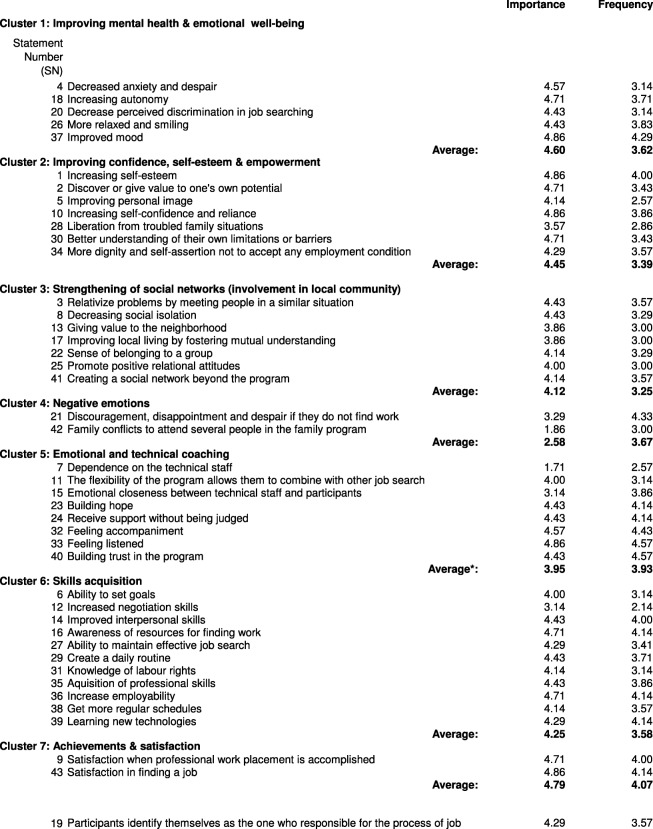
Statements and clusters resulting from group sessions held with technical staff and their scores based on criteria of importance, from [1] little importance to [5] extremely important and frequency of occurrence according to their professional knowledge, from [1] infrequent to [5] very frequent

*SN 7 in Cluster 5 was considered the only negative statement in the cluster. The average frequency and importance of the Cluster without this SN are 4.23 and 4.10, respectively

**SN 19 was related to the skills acquisition cluster for some of the participants and with improving confidence, self-esteem and empowerment cluster for some others. The final consensus was to leave the item in between these clusters.

According to the technical staff, the program had a positive effect on participants’ “Mental health and emotional well-being” (Cluster 1) by decreasing anxiety and despair (Statement Number, *SN*, 4), increasing autonomy (SN 18), and improving their mood (SN 37) (among others, See Table 3). It also improved their “Confidence, self-esteem and empowerment” (Cluster 2), and “Strengthened their social networks” (Cluster 3) by promoting positive relational attitudes (SN 25), and increasing the sense of belonging to a group (SN 22). Participants’ quality of life was also improved through “Acquisition of skills” (Cluster 6) and related items, including: a) technical skills, such as learning new technologies (SN 39) or professional skills (SN 35); b) daily life skills, such as the ability to set specific goals (SN 6), creating a daily routine (SN 29) and regular schedules (SN 38), and improving transversal competencies, among others; and c) job-seeking skills, such as awareness of job-seeking resources (SN 16), increased negotiation skills (SN 12), awareness of their labour rights (SN 31), as well as increasing employability (SN 36). Increased employability was located near the cluster entitled “Achievements and satisfaction” (Cluster 7). Technical staff also considered that EiN improved participants’ quality of life through “Emotional coaching and personalized technical assistance” (Cluster 5). They perceived that participants feel accompanied (SN 32), and appreciate that someone listens to them (SN 33) and offers support without judging them (SN 24). Finally, there was also a cluster of negative impacts for participants’ well-being, “negative emotions” (Cluster 4), which mainly resulted from frustration in not getting a job (SN 21).

The most important clusters, as rated by technical staff, were Cluster 1 (improved mental health and emotional well-being) and Cluster 7 (achievement and satisfaction). The clusters rated as most frequent were Cluster 5 (emotional coaching and personalized technical assistance, after excluding SN 7, “dependence on technical staff”), and Cluster 7 (achievement and satisfaction).

During the final group discussion, also known as participatory interpretation, which was only carried out with the group of professionals, the research team suggested that two types of clusters could be interpreted from the data analysis. This first type of clusters is those related to the specific program objectives (acquisition of skills, emotional coaching and personalized technical assistance, and achievements), and second type is those related to other effects of the program, such as positive impacts on mental health and emotional wellbeing. In contrast to the research team’s perspective, technical staff considered that, although the general objective of EiN is reemployment, the program’s specific aims were to improve participants’ confidence, self-esteem, and empowerment through emotional coaching and specific activities, as a path toward other employment insertion activities and increasing employability. Note that EiN targets groups with significant difficulty in labour market insertion, such as people at risk of social exclusion and those in long-term unemployment, some of whom are recruited through various social agents in the community, such as social services and street educators. Thus, some of the participants are in need of more extensive assistance, beyond reemployment. In this respect, any improvement in our target population should be considered a success.

## Discussion

The results obtained using the concept mapping methodology have allowed us to identify different mechanisms and effects by which the “Employment in the Neighbourhoods” ALMP can influence the quality of life of participants from the most deprived areas of Barcelona according to their perceptions and those of professionals. The effects described were perceived improved mental health and emotional wellbeing. Improved mental health has also been reported following a pre-post evaluation using quantitative methodology [8]. Participants’ also described negative emotions such as frustration and mood disorders linked to their failure to find a job or the cessation of subsidized employment.

Some of the suggested mechanisms for the perceived improvement on mental health and wellbeing were in line with Jahoda’s theory [9], namely strengthened social networks, daily structure, activity, and social identity. Some other mechanisms were in line with Fryer’s theory of increasing sense of control [13], namely increased empowerment, self-esteem, and confidence. One of the particularities of this study is that empowerment is manifested through increasing negotiation skills and the awareness of labour rights, in addition to increasing several skills which has been suggested by other studies [12]. This opinion was shared by professionals and participants of EiN, although further developed by professionals.

A somewhat new psychosocial factor highlighted by both professionals and participants was the feeling of accompaniment. One of the specific characteristics that differentiate EiN from other ALMPs, even in Barcelona, is the use of a personal guidance counsellor to accompany participants through the entire process. These results are consistent with those of previous qualitative research, where the attitude of personnel was essential, and where participants felt strong support from staff [17] and perceived them to be welcoming and non-judgmental [31], ideas that also appeared in our study. The reported consequences were not feeling alone, improved self-confidence and self-esteem, and increased optimism and joy [31]. Social support has been suggested as an important health-protecting factor among the unemployed [32]. EiN provided social support through both the technical staff’s support and a strengthened social network.

Finally, economic stability, as a material pathway, was highlighted by individuals who took subsidized employment, which also had a negative facet, namely its short-term duration.

Although we observed mostly positive perceptions related to quality of life, the quantitative pre-post intervention study evaluation showed that self-perceived health status remained stable or worsened [8]. Few studies have analyzed the effect of ALMPs on self-perceived health status, and those that have done so also found no statistically significant changes in self-perceived health status following the program [33–35]. Our first hypothesis is that changes in self-perceived health could take more time to emerge. EiN had a maximum duration of 1 year, and most of participants were only spending a few weeks or months. The post-intervention questionnaire was carried out one year after the beginning of the intervention.

Our second hypothesis is that individuals assess self-perceived health as a combination of information from specific health problems, general physical functioning and health behaviors [36]. Although the WHO definition of health as “a state of complete physical, mental and social well-being and not merely the absence of disease and infirmity” would categorize self-perceived health as an indicator of health-related quality of life [37], individuals assess self-perceived health as physical health. Physical health is much more difficult to change than mental health due to adaptive disorders.

### Gender differences of ALMPs on health

Although there is extensive literature on the differential effects of unemployment between genders, less is in known about the gender differential effects of ALMPs on health. One of the few published studies, a cross-sectional study from the UK, showed that life worth and happiness was higher among employed than unemployed male ALMP participants, and among females, participation had a marked effect on life satisfaction [5]. Rojdalen et al. [38] also designed a non-experimental study based on retrospective judgments in Sweden, taking gender differences into account. Mental health was more often judged by women and less well-educated people as being positively effect of training. Yet neither of these studies provided a sociological reflection on the mechanisms. A qualitative study among women from East London [39], where most programs are compulsory, showed that due to the temporary nature of the programmes, participants experienced feelings of insecurity, financial strain, role conflicts, and concerns about non maternal childcare. Mothers felt guilty for leaving their children during the training.

In our study, the women related their improvement to the ability of the program to reactivate their social network, to their perceived increase in self-esteem and confidence through the learning process, and to their feeling of accompaniment and protection by the technical staff. However, they also highlighted the health disadvantages of entering the labour market, given the health impacts of precarious employment, especially long working hours, which could enter into conflict with their family responsibilities. On the other hand, men related their improvement to the greater ability to find a job. Economic stability is recognized as one of the most important pillars for men’s well-being, and they highlight their job as a source of identity, again highlighting the breadwinner role. This appears to be specific to Spanish social context, and as we hypothesized, could be attributed to gender roles and the configuration of the family in Spain. No less important is the fact that both men and women mentioned how the ALMP changed their family relationship, which highlights how important it is for ALMPs in this specific context to account not only for the individual but also the relationship with the family, in order to be more effective, at least in terms of quality of life.

### Labour market situation

In some circumstances, programs that promote reemployment are not feasible, either because of a lack of jobs due to economic conditions or a lack of skills in specific disadvantaged populations. The Spanish labour market is characterized by persistent high unemployment and precarious employment conditions, including more temporary work, low salaries, vulnerability, and inability to exercise rights [40]. Labour market reforms implemented during the last years of the current economic recession have worsened this situation, increasing social inequality. Immigrants and manual and poorly-educated workers, as well as women and young people, are overrepresented in the most precarious jobs [41], and these groups are the target population of EiN. In this scenario, the program highlighted negative emotions of frustration and discouragement when participants were not reemployed, or reemployed in precarious jobs. These negative emotions emerged as a specific cluster in both the technical staff and participants groups, and were more important in the latter. In addition, finding a job in precarious employment improves wellbeing at first, but this is disrupted by the reality of precarious labour market conditions, or at the end of the 6–month term of jobs subsidized by the program. In this sense, one of the participants’ specific demands was that the program be continued, and that they maintain the counsellor or person of reference after completing the insertion itinerary. This suggests that the positive effects of EiN are probably not maintained beyond the end of the programme. However, this would be a hypothesis to be tested by means of a cohort study.

Considering labour market characteristics and health and wellbeing, we need to reflect on the notion that “any job is better than no job” [9]. The simplistic dichotomy between employment and unemployment is rather more complex than viewing unemployment as “bad” and employment as “good”. This is particularly pertinent given evidence that the labour market is often dominated by low pay and limited sustainability, such that reemployment in a precarious position could be more detrimental for health and quality of life [42]. Thus, there is a need for a comprehensive program for unemployed individuals, including economic benefits and activities to reintroduce them into the labour market. A comprehensive program could trigger both material and psychosocial mechanisms to cushion the effects of unemployment. These measures should be accompanied by effective policies to improve job quality.

### Strengths and limitations

Concept mapping is a strong method for analysing perceptions. Our study introduces the double perspective of professionals and participants, which enriches the analysis and highlights gender differences. Moreover concept mapping is very useful evaluation technique, as it can help to develop a model or theory for change. Theory of change explains how the activities undertaken by a program, such as an ALMP, contribute to a chain of results that lead to the perceived effects. Thus concept mapping enables us to evaluate a complex intervention and complements the quantitative assessment to better understand its results [8]. Finally, maps are easy to read and interpret by decision-makers and the general public. To our knowledge, this is one of the few health-related evaluations of ALMPs in Spain, and in other Southern European countries. Thus, our study makes an important contribution because, compared to Northern Europe, Southern European countries have different welfare systems and different gender and family regimes.

This study also has some methodological limitations, the most important one being the participants’ difficulty in reading comprehension and conceptualization skills due to their educational background, or because they were non-native speakers of Spanish or Catalan. Although this was not problematic for the brainstorming session, it was the main drawback during the rating and sorting session, in that some participants were not able to sort the statements with respect to their similarity to some overarching concept. This is reflected in the cluster map, in that the distance between statements in each cluster are longer in the participants’ map, than in the professionals’ one. Moreover, the use of a cut-off filter of 3 in the final map indicates the difficulty in interpreting it due to spurious relationships between statements. We suspect that there was a lot of noise in the sorts, and that it would be useful to re-compute maps filtering out these spurious relationships, leaving only the most frequently made associations.

Also, participants’ difficulty in reading comprehension and conceptualization skills was the main reason for not conducting the final group discussion with EiN participants, i.e. the participatory interpretation session. In this session, researchers shared different map solutions with participants, discussed their interpretation, and possible cluster names. This last step in the methodology, although not compulsory, increases the exchange of knowledge between researchers and participants. Thus, this last step was only conducted with professionals but not EiN participants. In this case, the research team choose the final map and the names of the clusters. On the other hand, the EiN participants showed extremely rich generation of ideas, most of which were consistent with the technical staff’s perceptions. After finalizing the study, we would recommend other qualitative techniques when working with this specific population; nonetheless, it was an excellent technique for working with professionals.

Regarding internal validity, while we emphasised the importance of discourse variability, our results may be prone to self-selection bias. Participants who decided to take part in the study could be those ones with the highest satisfaction, although it was not possible to control this, as no register of those who left the programme was available. Moreover, professionals may feel pressure to point out more positive effects than negative ones.

Regarding external validity, this study may not be representative for the entire population participating in EiN. The aim of the technique is to achieve maximum discourse variability to ensure that different conceptual meanings are collected. To accomplish this aim, we conducted theoretical sampling and ensured that socio-demographic characteristics were similar to those of the entire population. Moreover, we conducted two different concept mappings, one with participants and one with professionals, with similar results. However, a more complete concept to increase external validity might be achieved by repeating the same procedure several times, in different years, and with different groups. Note that discourse saturation was reached after conducting the four groups of ALMP’s participants.

### Recommendations

The mechanisms related to ALMPs is still an underexplored topic, especially from the epistemological perspective of situated knowledge, creating opportunities for challenging research. There are two immediate research challenges. First, based on the results of the concept mapping, we suggest conducting in-depth interviews with EiN participants in order to further explore the chain of mechanisms and triggers. It would be extremely useful to conduct a qualitative longitudinal study to follow participants from the moment before entering the program, at different cut-points during the program, and a few months after it ends. Second, using information from the qualitative study, it would be possible to construct a quantitative tool for measuring the mechanisms underlying ALMPs, and to validate this in terms of psychometric properties. This could be implemented in a quantitative longitudinal cohort to properly evaluate the mechanisms and effects of EiN and similar programs.

### Final remarks

The impact of ALMPs in relation to their costs is often questioned. Of course, the criteria for success of an ALMP depend on the theoretical perspective on which the evaluations are based. These criteria could be the degree of participation in activation programmes, which would increase participants’ chances of reemployment [43]. From a public health perspective, and from a “health in all policies” approach [44], improved health and wellbeing should also be one of the criteria for success. There is generally limited scientific evidence in this field, especially in relation to labour market policy-makers’ inclusion of health and wellbeing as a measure of the success of ALMPs. Clearly, there is a societal benefit to evaluating programs and policies, not purely in economic terms, but also in terms of health and wellbeing.

## Conclusions

The “Employment in the Neighbourhoods” program showed important advances in the quality of life of participants according to their perceptions and those of technical staff, mainly in terms of better perceived mental health and emotional wellbeing. The mechanisms for these perceived improvements were skills acquisition, strengthened social networks, emotional coaching and personalized technical assistance, and providing social support. Moreover, our study provides some insights into the strengths and weakness of the program, which would be useful for improving it.

## Supplementary information

**Additional file 1: Table S1.** Summary of actions in the “Employment in the Neighbourhoods” program

## Data Availability

The datasets used and/or analysed during the current study are available from the corresponding author on reasonable request.

## Abbreviations

ALMP: Active Labour Market Programme
GDP: Gross Domestic Product
MDS: Multidimensional Scaling
SN: Statement Number
