# Supplementary material for: How does a targeted active labour market program impact on the well-being of the unemployed? A concept mapping study on Barcelona “Employment in the Neighbourhoods”
Source: BMC Public Health. 2020 Mar 17;20:345. doi: 10.1186/s12889-020-8441-2 (PMC7076971; doi:10.1186/s12889-020-8441-2)
Supplement: Supplementary file 1 — Additional file 1: Table S1. Summary of actions in the “Employment in the Neighbourhoods” program [file 12889_2020_8441_MOESM1_ESM.pdf]

Table S1. Summary of actions in the "Employment in the Neighbourhoods" program

Action	Description
Informative Session	The purpose of this session was to explain the content and objectives of the program, while describing the resources available in the district and other cities for people seeking work and training.
Personal interview	The purpose of the interview is to collect information on the participant's socioeconomic, personal and family situation, and to evaluate their skills, and suggest a personal itinerary for job search and training.
Individual coaching	The frequency is based on the participant's need and the availability of technical staff. The content of each session depends on the participant's needs and their itinerary. Individual coaching is offered during the process of training, and is continued after participants have been inserted in the labour market.
Comprehensive itinerary 1: professional training	Transversal key competences, including several modules: 1) motivation and self-awareness, 2) communication and teamwork, 3) Information and Communication Technologies (digital basic skills), 4) Mobility (increasing autonomy and knowledge of use of public transport), 5) Insertion (working on the skills necessary to actively seek employment independently, and acquisition of skills to perform successful selection interviews).
Comprehensive itinerary 2: job searching skills	Various workshops: 1) how to write a CV and a cover letter, 2) Analysis of Employability, 3) Analysis of the labour market, 4) Registration in job search portals, 5) How to organize my job search, 6) Job search channels, 7) How to do a job interview, 8) Workshop for youth seekers, 9) Workshop on the Barcelona Activa Platform, 10) Labour market sectors with greater opportunities to be employed.
Job search area	Provides computers, internet conncection, and supervision from coaching staff.
Vocational training	Includes work-placement activities
